# Transgenerational Inheritance of Diet-Induced Genome Rearrangements in Drosophila

**DOI:** 10.1371/journal.pgen.1005148

**Published:** 2015-04-17

**Authors:** John C. Aldrich, Keith A. Maggert

**Affiliations:** 1 Department of Biology, College of Science, Texas A&M University, College Station, Texas, United States of America; 2 Department of Cellular and Molecular Medicine, College of Medicine, University of Arizona, Tucson, Arizona, United States of America; Centre National de la Recherche Scientifique, FRANCE

## Abstract

Ribosomal RNA gene (rDNA) copy number variation modulates heterochromatin formation and influences the expression of a large fraction of the Drosophila ge-nome. This discovery, along with the link between rDNA, aging, and disease, high-lights the importance of understanding how natural rDNA copy number variation arises. Pursuing the relationship between rDNA expression and stability, we have discovered that increased dietary yeast concentration, emulating periods of dietary excess during life, results in somatic rDNA instability and copy number reduction. Modulation of Insulin/TOR signaling produces similar results, indicating a role for known nutrient sensing signaling pathways in this process. Furthermore, adults fed elevated dietary yeast concentrations produce offspring with fewer rDNA copies demonstrating that these effects also occur in the germline, and are transgenera-tionally heritable. This finding explains one source of natural rDNA copy number variation revealing a clear long-term consequence of diet.

## Author Summary

We show that altering the nutritional medium of Drosophila cultures, emulating dietary largess in the wild, increases expression of the high copy-number ribosomal RNA genes and results in rDNA instability and loss. The reduction in gene copy number occurs both in somatic and germ cells, such that altered copy numbers are transmitted to the next generation. Our findings have clear ecological and disease relevance. The reduction of supernumerary ribosomal RNA gene copy number has been previously shown to compromise both epigenetic and genomic stability and alter the expression of hundreds of genes, while rDNA instability itself is a key progression hallmark of some cancers. This study links diet and Insulin/Insulin-like Signaling modulation to changes in the genome and provides an accounting for natural copy number variation of rRNA genes.

## Introduction

It is clear that an organism’s gene expression patterns are responsive to environmental input. Often, this influence is not limited to short-term regulatory changes, but can persist through multiple cell divisions and can, in some cases, be transmitted to offspring. Typically, such changes are identified as “epigenetic” and are thought to be mediated by a variety of chromatin modifications [1–8]. However, because genome stability, particularly of highly-repetitive (e.g., pentameric repeat) sequences or middle-repetitive transposable elements, is modified by silencing involving repressive histone modifications, “epigenetic” perturbations may have both direct and long-term consequences: the former caused by disruption of silencing leading to “epigenetic instability,” and the latter by creating transmissible changes to chromosomes that themselves may affect gene regulation in subsequent generations. This consideration significantly adds to models of epigenetic inheritance that often overlook the ease with which histones and DNA methylation can be modified and the rapid rate at which they are turned over in non-dividing cells [9, 10]. Recent [11–14] and previous [15] findings show epigenetic silencing is unstable even in non-dividing cells, making it a particularly difficult challenge to reconcile models of chromatin (e.g., histone) mediated epigenetic silencing with transgenerational (i.e., mitotic and/or meiotic) inheritance.

*35S* Ribosomal RNA gene (rDNA) transcription has been a powerful model for understanding the regulatory effects of chromatin modification because evidence suggests identical genes may adopt different stable activity states (expressed versus repressed) even when immediately juxtaposed [16–19]. Transcription from tandem repeated rDNA arrays accounts for approximately 50% of total transcription [20] and is regulated such that only a subset of the redundant copies are active in a given cell type, while the remainder are inactive and are accompanied by chromatin structure consistent with silencing [21, 22]. Consequences of misregulation are severe, in part due to the tandem repeat of identical sequence. Mutations in silencing factors (*e*.*g*., gene products of the *dcr-2*, *Su*(*var*)*3-9*, *HP1*/*Su*(*var*)*205*, *PARP*, *CTCF*, and *sir2/sirt* loci) result in supernumerary mini- or micro-nucleoli and rDNA copy number reduction [23–27], possibly through increased frequency of intrachromosomal recombination resulting from the repair of transcription-induced damage [28, 29]. The tendency for natural loss and the ability of some rDNA arrays to expand through unknown processes [30–32] contribute to striking variation in rDNA copy number in both wild and laboratory strains [33–36]. This variation, in turn, is a potent genetic modifier of a number of phenomena, including the regulation of ecologically- and metabolically-relevant gene networks, the stability of genome structure and heterochromatin silencing, stress responses, and potentially metabolic function [24, 26, 37–46].

The relationship between rDNA transcriptional activity and rDNA array stability suggests a non-epigenetic mechanism through which the environment might induce heritable and consequential changes in the genome through long-term (i.e., permanent) modulation of genetic variation and epigenetic stability. Although the change may bear the hallmarks of epigenetic regulation (*i*.*e*., inducible, heritable, consequential), it may not technically be considered epigenetic because it involves chromosome changes [47]. Nonetheless, because rDNA copy number modulates the stability of epigenetic silencing [24, 26, 43], the origin of rDNA copy number variation is a significant concern to studies of “hidden” regulatory variation, heterochromatin, and epigenetics.

The aim of this study was to identify a natural, ecologically-common, and human health-relevant source of rDNA copy number variation. Expanding on previous work suggesting that an increase in rDNA expression may lead to its loss, we hypothesized that rDNA copy number changes can be induced by modulating diet. In support of our hypothesis, we found that flies raised on high dietary yeast concentration had increased supernumerary nucleoli as larvae, and somatic rDNA copy-number reductions as adults. Similar results were observed in flies expressing a hypermorphic insulin receptor allele, and were reproduced pharmacologically using human insulin *in vitro*, suggesting that diet-induced rDNA instability is mediated by known nutrient signaling pathways. Drugs that inhibit expression of the rDNA mitigated instability and loss, suggesting the effects were a consequence of expression. Furthermore, adult males fed high-yeast diets produced offspring with fewer *Y*-linked rDNA copies demonstrating that the effect was transgenerationally heritable. These findings identify diet as a potential source for rDNA variation observed in natural populations and suggest a mechanism through which environmental conditions might result in induced “transgenerational” genome changes.

## Results

Previous work from yeast and filamentous fungi, Drosophila, plants, and experimental mammal systems have suggested that rDNA expression increases in response to diet, while other work has shown that derepression of the rDNA results in instability and loss. Together, these imply that natural rDNA variation may be reflective of the nutritional history of a population. Because of the growing awareness of the importance of rDNA instability and copy number variation in phenotypic variation, stress response, and disease, we directly tested our hypothesis that rDNA copy number may be manipulated by affecting diet.

### Dietary manipulation

Dietary composition is an easily modified environmental variable in Drosophila laboratory studies and has been shown to influence a variety of complex phenotypes including rRNA transcription and ribosome biogenesis [48–51]; we therefore considered it as a potential source of “natural” rDNA instability and variability. In this study we used two experimental media based on those used in dietary restriction studies [52], consisting of a constant carbon source (5% sucrose) and altered concentrations of nutritional yeast (10%, and 30% w/v). Throughout the study we refer to these as SY10 and SY30 respectively (Sugar-Yeast-w/v). Larvae raised on SY30 developed to pupation approximately 9 hours earlier than their SY10 raised counterparts. Apart from this observation, there were no obvious differences in terms of body mass or survival to adulthood between the two media. To obviate secondary effects on culture conditions based on crowding, and to assure similarity in conditions as much as possible, we controlled density of eggs, larvae, and adults in vials by collecting eggs from petri dishes containing agar made from apple juice, suspending eggs in PBS, and pipetting identical volumes of eggs to SY10, SY30, or standard cornmeal medium.

### Dietary manipulation alters rDNA expression and rDNA stability

**Fig 1:**
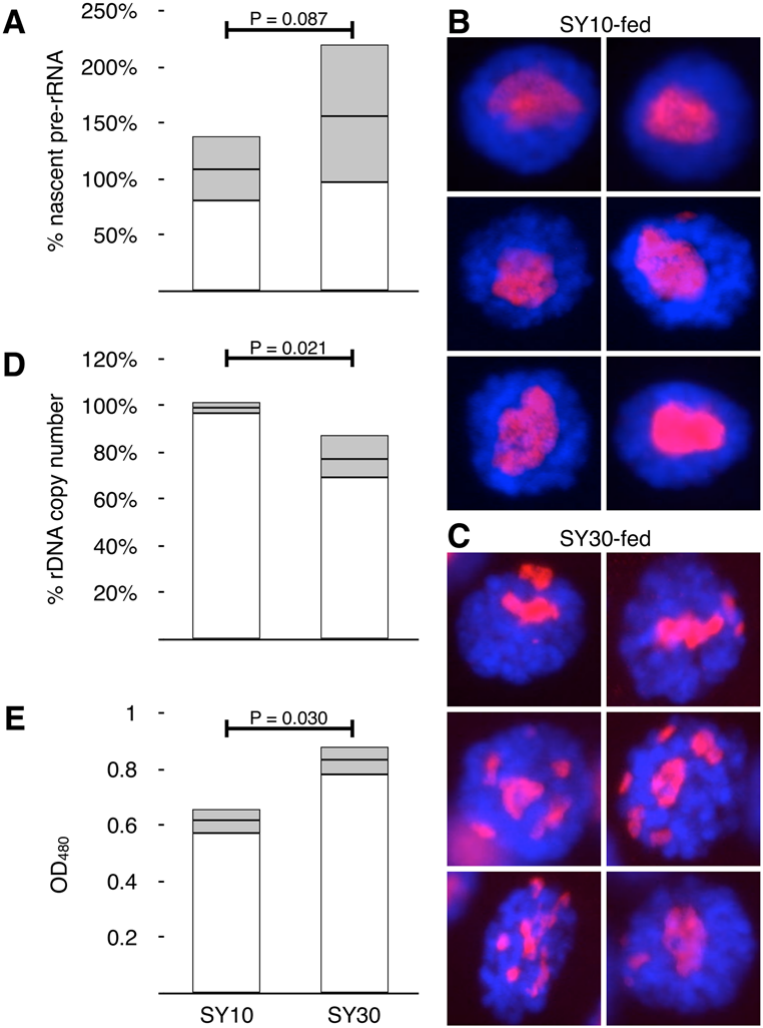
Larval diet influences rDNA activity and stability. **(A)** Real time PCR quantification of cDNAs derived from unprocessed (ETS-18S junction) pre-rRNA from larvae fed either SY10 or SY30 diets. Values were normalized to the genomic DNA copies of tRNA^K-CTT^ genes and proportions plotted relative to Standard-fed larvae (defined as 100%). Error bars report standard deviation of RNA quantities derived from five independent pools of larvae for each condition, and indicate differences between populations exposed to altered dietary source. Although population distributions (shown) overlap, average rRNA expressions of SY10 vs SY30 differ. **(B)** Gallery of representative salivary gland nuclei obtained from SY10-fed larvae. Frequency of nuclei with multiple nucleoli was 7% ± 6% (S.D.), N = 337. α-Fibrillarin stains nucleoli red, DAPI stains DNA blue. **(C)** Gallery of representative salivary gland nuclei obtained from SY30-fed larvae, stained as in (B). Frequency of nuclei with multiple nucleoli was 40% ± 24% (S.D.), N = 522. **(D)** Real-time quantitative PCR analysis of *35S* rDNA copy number in adult males raised on SY10 or SY30 as larvae. Percentages calculated relative to isogenic flies raised on standard food (defined as 100%). Error bars are standard deviation of three independent biological replicates and 3–4 technical replicates of each, and so contain pooled standard deviations of the populations and standard errors of the quantification. **(E)** Quantification of acidified-alcohol-extractable pigment from *white* ^mottled-4^ flies raised on SY10 and SY30. Error bars are standard deviation of three parallel biological replicates each containing heads from 20 individuals. All P-values (in (A), (D), and (E)) were calculated using Student’s t-test.

We fist confirmed that altered diet affected rRNA expression. We could discriminate accumulated mature rRNA products (*18S*, *28S*, *5*.*8S*), for instance bound in ribosomes, from actively-transcribed pre-rRNAs (*35S*, or *45S* in some organisms) by detecting the quantity of cDNA derived from the pre-processed 5’-most sequence of the *35S* primary transcript containing the External Transcribed Spacer (ETS). The ETS is constitutively processed during maturation of the pre-rRNA *35S* transcript and quickly degraded, and is therefore used to measure *de novo* rDNA expression [26, 53, 54]. We compared male flies of genotype *yellow* ^1^ *white* ^67c23^/*Dp(1;Y) y* ^*+*^, *P{w = RS5}10B* (henceforth *Y*,*10B*), upon which we have performed other studies of the ribosomal rDNA [24, 41, 55–58]. We detected an approximately 50% increase in pre-rRNA levels in populations of second instar larvae raised on SY30 compared to Standard media (Fig 1A), confirming the suitability of these media for this study.

We addressed whether an increase in dietary yeast concentration during development would result in rDNA loss. In interphase cells, the rDNA is the genetic location of the cytogenetic Nucleolus Organizing Region (NOR), and thus the foundation of the nucleolus. Even single rRNA genes are capable of forming tiny supernumerary nucleoli [59]. Consequently, nucleolar morphology is sensitive to the overall size, activity level, and integrity of the rDNA arrays. In Drosophila, rDNA damage is readily observed in larval salivary glands. Damage and repair are thought to lead to extrachromosomal circles, which coalesce mini- or micro-nucleoli in non-dividing or post-mitotic cells, forming supernumerary nucleoli. Such “fragmentation” has been observed in flies mutant for “heterochromatin components” [23–26], where it is thought to stem from aberrant intrachromosomal recombination resulting in the formation of extrachromosomal rDNA circles [28, 60]. We performed immunofluorescence on larval salivary gland cells with an anti-fibrillarin antibody to detect the nucleolar dense fibrillar component which, in Drosophila, typically forms a single focus containing both *X*-linked and *Y*-linked rDNA arrays. We observed an elevated frequency of multiple nucleoli in SY30-fed larvae compared to SY10-fed larvae (Fig 1B and 1C). Multiple nucleoli (defined as more than one discrete separate fibrillarin focus) were present in 40% ± 24% of the nuclei within single salivary glands dissected from SY30-fed larvae; in contrast multiple nucleoli were observed in 7% ± 6% of salivary gland nuclei from from SY10-fed larvae. The ranges are standard deviations of nucleolar instability rates from independently grown and dissected salivary glands, indicating that populations of genetically-identical individuals raised on different food sources show variation that can be discriminated by their averages.

In dividing cells, acentric extrachromosomal rDNA circles are lost, effectively reducing rDNA copy number and shortening the rDNA array through development. To quantify rDNA loss stemming from diet-influenced extrachromosomal circle formation during development, we used real-time PCR to measure the rDNA abundance of flies raised as larvae on either SY10 or SY30. Relative copy number was quantified using genomic copy number of a tRNA gene as normalization resulting in a DNA-to-DNA proportion [55]. rDNA copy number differences were monitored by comparing the rDNA copy number in freshly eclosed F1 males to that of males taken from the “F0” parental stock (i.e., treated males compared to their fathers and uncles). Male adults were collected within hours of eclosion, thus any diet-influenced changes to rDNA copy number were the result of physiological effects initiated prior to metamorphosis. The rDNA copy number of flies raised on SY10 was indistinguishable from that of their sires, while those raised on SY30 exhibited an average copy number reduction of ~20% (Fig 1D). The standard deviation for these data are derived from independent biological replicate vials from sibling males. The standard deviation of the F0 (not presented on graph, but whose average is defined as 100%) is pooled into the SY10 and SY30 populations.

It has been known that culture conditions affect the extent of position effect variegation (PEV), the somaclonal variation that occurs at new juxtapositions of euchromatin and heterochromatin. Specifically, richer or more abundant food leads to reduced heterochromatin-induced gene silencing and thus higher levels of variegating gene expression [61, 62]. To address whether SY10 and SY30 alter the extent of PEV, we raised isogenic *white* ^mottled-4^ (*w* ^m4^/*Y*,*10B*) males on SY10 or SY30. The only source of *white* ^+^ expression in this genotype is from the *white* ^mottled-4^ allele. If SY30 reduced silencing, we expected to see increased eye pigmentation. Three days after eclosion, we decapitated males and extracted pigment from their heads in acidified alcohol. The extent of the suppression of variegation, observed as increased pigment extraction (Fig 1E), accords well with the increased expression as a result of reducing rDNA copy number through molecular means [24, 55] or from wild-caught strains [43, 63].

The quantified loss of rDNA due to SY30 is within the range of natural *Y*-linked rDNA variation [35, 55, 64], and within the experimental range used to demonstrate heterochromatin changes and gene regulatory variability by us and others [24, 64] on the Y,10B chromosome specifically. Therefore, altered diet could in principle be responsible in entirety or in part for the natural rDNA copy number variance observed in natural populations isolated from the wild.

### Diet-induced rDNA loss is independent of R1 and R2 expression

In Saccharomyces, it is thought that rDNA damage and loss is a consequence of a collision between the replication fork and transcriptional machinery at the rDNA array [29]. This model accounts for the transcription-dependent nature of rDNA recombination as well as the relationship between rDNA stability, cell division, calorie-restriction, and aging [27, 50, 65]. Much less is known about the regulation of rDNA stability in multicellular organisms and, therefore, the precise mechanism underlying our observations is unknown.

There is no known analogous system for specific loss in Drosophila (or any of the metazoa), however the unique retrotransposable elements in arthropods provides one possibility. It is possible that loss of rDNA in Drosophila is a direct consequence of activity, for instance derepression of the rDNA leading to mobilization of *R1* or *R2* retrotransposons, whose mobilization is known to cause single-stranded DNA breaks. This possibility makes two predictions that we tested.

**Fig 2:**
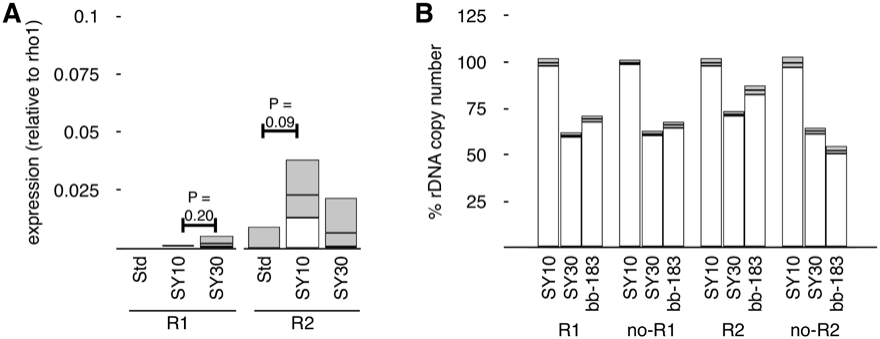
Copy number and RNA expression of *R1* and *R2* retrotransposable elements are not discordant. **(A)** Reverse Transcriptase Real-time PCR of expression of *R1* and *R2* transcripts from flies raised on Standard, SY10, or SY30 food sources. Values are normalized to *rho1* mRNA and relative to *R1* and *R2* RNA levels in flies raised on Standard food, errors indicate standard error of the mean and capture increases in pooled populations raised on different food sources. Expression levels do not differ significantly from each other (only P-values less than 0.25 are shown). **(B)** Copy number determination of *R1* and *R2* elements in the *Y*-linked rDNA loci of flies raised on SY10, raised on SY30, or a rDNA deletion allele from a previous study with *I*-*Cre*I-mediated rDNA loss (*bb-183*). “R1” detects unique *R1*-rDNA junctions, while “noR1” detects the rDNA flanking the stereotyped *R1* insertion site in the rDNA. “R2” and “noR2” similarly detect *R2*-inserted *35S* rDNA and *35S* without *R2* insertion, respectively. Note that each reaction uses separate primers, so comparisons between primers (“R1,” “noR1,” “R2,” “noR2”) are not valid, while comparisons between treatments (SY10, SY30, *bb-183*) are.

First, we investigated whether consumption of SY30 lead to pronounced derepression of *R1* or *R2*, the retrotransposable elements resident in the Drosophila rDNA. We could not detect significant increases in expression of either when raised on SY10 or SY30 (Fig 2A). Second, we investigated whether *R1*- or *R2*-interrupted *35S rRNA* genes were preferentially lost after growth on SY30. We observed decreased R1-interrupted and R2-interrupted rDNA copies, as well as loss of uninterrupted *35S* rDNA copies, but neither R1 nor R2 reductions were strongly biased over decreases as a result of *I*-*Cre*I-induced damage (Fig 2B) [55].

It should be noted that absolute numbers of *R1*- and *R2*- containing *35S* genes are not possible to reliably calculate, nor are our “R1” and “no R1,” or “R2” and “no R2,” numbers necessarily additive, because these data are derived from different priming real-time PCR oligonucleotides, thus do not have the same kinetics of amplification [55]. Nonetheless, comparisons between conditions are valid, and in these cases we see no strong biased loss of R1 or R2 sequences (judged by either *R1*- and *R2*-containing or *R1*- and *R2*-lacking rRNA genes). We cannot specifically quantify rRNA genes with both insertions (although these likely represent a vast minority of rRNA genes [66, 67]), or that are free from either retrotransposable element insertions (likely the vast majority). Together these data argue against an *R1*- or *R2*-related mechanism of chromosome damage and rDNA loss. Work by Cohen and colleagues additionally showed that repetitious gene arrays that do not house such retrotransposons show progressive loss through the lifespan of Drosophila [28], similar to the rDNA and similar to what we observe in SY30 conditions.

However it requires extreme caution in interpreting these results. We know that SY30 causes about 20% reduction in total rDNA copy number, and the rate, extent, and cytological manifestation of this loss indicate that rDNA loss is not of individual rRNA genes. Instead the best inference is that large contiguous blocks of rRNA genes—containing *35S* genes interrupted and uninterrupted by *R1*, *R2*, or both—are lost as groups. The arrangements of the inserted rDNA genes is thought to vary considerably [18], so recombination, damage-and-repair, or loss events that occur between non-juxtaposed rDNA genes may remove a considerable number of rRNA subtypes (uninterrupted, *R1*-interrupted, *R2*-interrupted, and double-interrupted) that themselves were not part of the mechanism of loss.

### Altered insulin receptor activity (insulin/insulin-like signaling) affects nucleolar stability

**Fig 3:**
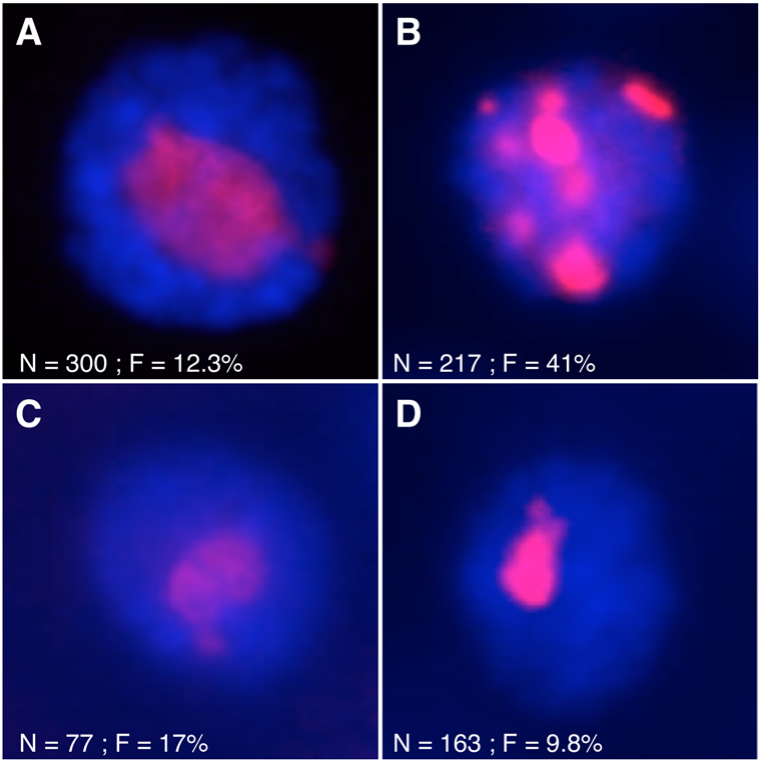
Mutations affecting nutrient signaling perturb nucleolar stability. **(A)** α-Fibrillarin immunofluorescent detection in salivary gland nuclei from wild-type larvae or **(B)** larvae expressing a hypermorphic insulin receptor allele (InR.R418P) under the control of Ubi-GAl4. **(C)** Flies expressing InR.R418P were raised on food tainted with 10 µM rapamycin did not exhibit multiple nucleoli. **(D)** Flies expressing an antimorphic insulin receptor allele (InR.K1409A) did not have multiple nucleoli. In all images, red shows fibrillarin and blue shows DNA. N = total number of nuclei scored, F = percentage of nuclei with multiple nucleoli.

If instability is a consequence of changes in nutrient availability, then we reasoned that modulating known nutrient signaling pathways should produce similar results. In Drosophila, as in *Cænorhabditis elegans*, mouse, and human, the insulin/insulin-like growth factor signaling (IIS) and TOR signaling networks mediate many cellular responses to nutrient availability, including ribosome biogenesis and rDNA expression [49, 68, 69]. We expressed a constitutively active form of the insulin receptor (InR.R418P) either generally in larvae (using a Ubiquitin promoter to drive GAL4 expression in all cells) or specifically in salivary glands (using the Sgs3 promoter to drive GAL4 expression in larval salivary glands). Because these cells do not undergo cytokinesis, rDNA removed from the chromosomal rDNA array (e.g., as extrachromosomal circles) would not be measurably lost using real-time PCR, thus we used the formation of supernumerary nucleoli as proxy for rDNA instability [23, 25]. In both cases we observed elevated levels of multiple nucleoli (~41% and 28% respectively) (Fig 3A and 3B), indicating that activating the Insulin Receptor was sufficient to induce instability. Raising flies expressing constitutive InR.R418P on food laced with rapamycin mitigated the effect (Fig 3C), suggesting inhibition of rDNA transcription suppressed the nucleolar instability. Expression of an antimorphic allele (InR.K1409A) had no apparent effect (Fig 3D), indicating that either antimorphic receptor expression levels were insufficient to reduce rDNA expression and affect loss rate, or the basal level of nucleolar instability we observe is independent of IIS-mediated rDNA expression.

### Acute loss of nucleolar stability can be effected by insulin treatment in vitro

In order to limit our view to acute cell-autonomous effects of nutrient signaling perturbation, we next opted to modulate the activity of these pathways pharmacologically in cultured larval salivary glands, a fully developed, post-mitotic tissue. In this way we could separate developmental defects (as a result of prolonged expression of hypermorphic or antimorphic alleles) from acute defects caused by altered cell physiology.

**Fig 4:**
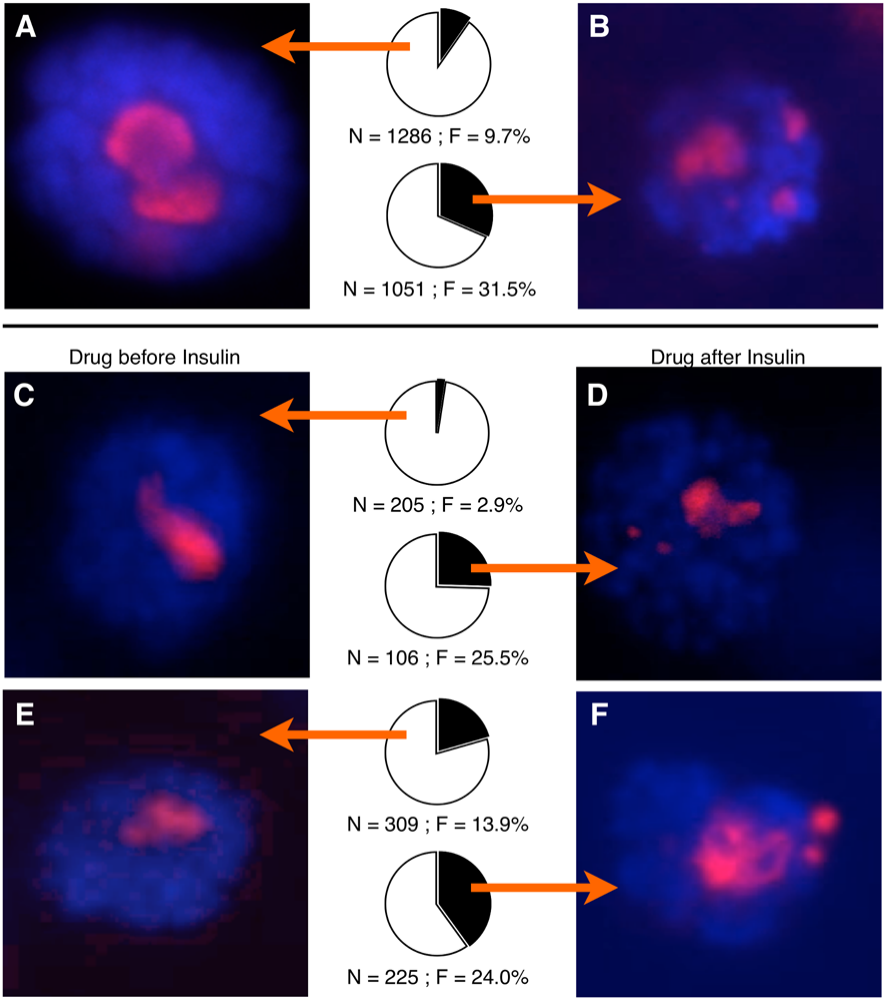
Pharmacological insulin treatment acts acutely to destabilize nucleoli. **(A)** Representative picture of a cultured salivary gland expressing a mRFP-Fibrillarin fusion gene and counterstained with DAPI. Pie chart shows percent of nuclei that had single nucleoli (white) or multiple nucleoli (black), and arrows indicate which population of nuclei images were taken from. N = total number of nuclei scored, F = percentage of nuclei with multiple nucleoli. **(B)** As in (A), but cultured with recombinant human insulin for 24 hours. **(C)** As in (B), but with a 2-hour treatment of Rapamycin prior to insulin addition. **(D)** As in (B), but with a treatment of Rapamycin during the last 2 hours of insulin exposure. **(E)** and **(F)** are as (C) and (D), respectively but treatment was with Actinomycin-D instead of Rapamycin.

We dissected larval salivary glands from flies expressing a Fibrillarin-RFP fusion protein [70] whose expression was controlled by a heat shock responsive hsp70 promoter. We did not induce expression with heat shock because sufficient nucleolar RFP was detectable without heat shock. Salivary glands were cultured in Drosophila cell/tissue culture medium for 22–24 hours in the presence (or absence) of recombinant human insulin. Treatment with insulin resulted in increased supernumerary nucleoli in live salivary glands (Fig 4A and 4B). Exposure to either actinomycin D or rapamycin (two drugs which block RNA Polymerase I activity, the former directly and the latter by inhibiting TOR) for two hours prior to insulin addition abrogated the multiple nucleolar morphology (Fig 4C and 4E). This pharmacological suppression of supernumerary nucleoli could not be reproduced if the drug was administered in the last two hours of insulin exposure (Fig 4D and 4F), supporting the interpretation that drug-induced stability of the nucleolus was a consequence of reduced rDNA transcription, rather than a reorganization of the nucleolus as a result of drug exposure. We confirmed that drug treatment reduced active rRNA expression by culturing eviscerated whole wild-type larvae in tissue culture medium for 24 hours in the presence of rapamycin or actinomycin D alone. Real-time PCR quantification of cDNAs of pre-processed rRNA junctions (as in Fig 1A) were reduced to 80% (+19.8%/-15.9%) and 69% (+13.1%/-11%) (N = 10 larvae for each condition), respectively, compared to control larvae cultured without any drug. That nucleolar instability is enhanced by insulin and mitigated by rapamycin and actinomycin D suggests that the consequential effect on rDNA stability occurs downstream of the convergence of the activities of these pharmacological agents.

### Loss of rDNA manifests on meiotically-inherited chromosomes

**Fig 5:**
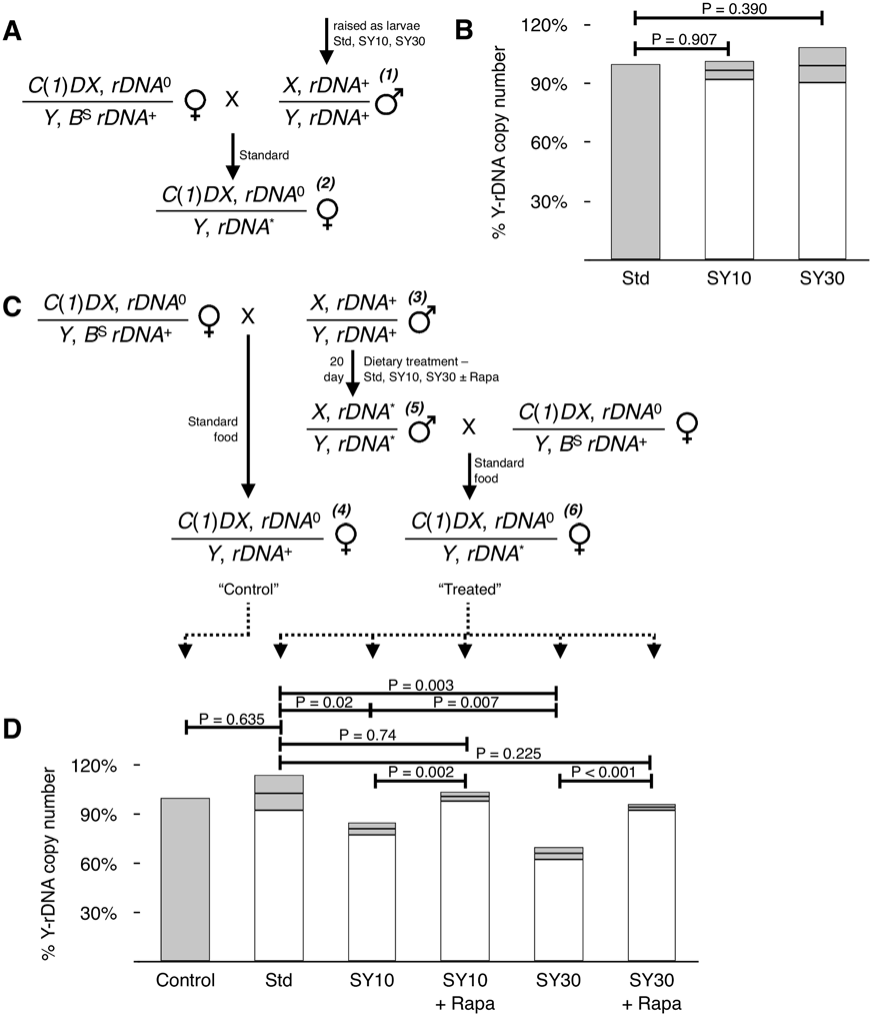
Adult diet changes rDNA copy number of progeny. **(A)** Crossing scheme used to genetically isolate *Y*-linked rDNA arrays for quantitative real-time PCR analysis. DNA was extracted from female progeny. *C*(*1*)*DX* has no rDNA (rDNA^0^), *X* and *Y* chromosomes initially have normal complements of rDNA (rDNA^+^), and effects of diet are assessed on the patroclinous *Y*-linked rNA (rDNA*) Numbers (***(1)*** and ***(2)***) are referred to in the main text. **(B)** *Y*-linked rDNA copy number of the progeny of males raised on SY10 or SY30 as larvae. Percentages calculated relative to the progeny of males raised on Standard (Std) food, defined as 100% (gray bar). N = 3 pools each of 20 larvae for each condition. **(C)** Crossing scheme used to treat and isolate *Y*-linked rDNA arrays for analysis. “Control” flies were derived from freshly-eclosed males mated to *C*(*1*)*DX* females and raised entirely on Standard food, and dietary-manipulated flies were derived from crosses of the same males after brooding on different food sources (see text, including references to ***(3)***-***(6)***) then outcrossed to *C*(*1*)*DX* females and the progeny raised entirely on Standard food. **(D)** *Y*-linked rDNA copy number of progeny of adult males kept for 20 days on SY10, SY30, or Standard food with or without 10 μM Rapamycin. Percentages calculated relative to the progeny of males mated prior to the 20-day treatment (“Control” in (C)). Error bars are standard deviation of three independent biological replicates each of ten sibling females. P-values calculated using Student’s t-test.

Wild-caught Drosophila strains exhibit a wide variance in rDNA copy number [34, 35]; the source of this variance, however, is unknown. For this variability to be explained by environmentally induced instability during the life history of these chromosomes, germline rDNA would have to be susceptible to environmental influence. To look for possible germline effects of diet, we used a genetic strategy to specifically measure copy number of *Y*-linked rDNA. We chose to focus our attention on the *Y*-linked array because it is preferentially active in males [43, 71] and because of the ease with which the *Y* chromosome is manipulated genetically [55]. We genetically-isolated *Y*-linked rDNA arrays by crossing adult males to females bearing an rDNA-deficient compound *X* chromosome (*C*(*1*)*DX*) (Fig 5A). Female progeny of this cross were viable and carried the patroclinous *Y*-linked rDNA as their sole source of rRNA genes; any differences between rDNA in daughters were due to permanent germline changes to the chromosomes occurring in the fathers.

When male flies were raised as larvae on either “Standard” cornmeal medium (“Standard” or “Std”), or SY10 or SY30 (Fig 5A, symbol ***(1)***) then moved to Standard food and outcrossed to *C*(*1*)*DX* virgin females, the progeny (***(2)***) had no detectable difference in the rDNA copy number (Fig 5B). Thus while the soma was undergoing diet-induced rDNA loss at this stage (Fig 1D), the germline was not susceptible to diet induced loss of rDNA; this was not unexpected because the germline cells are relatively quiescent in larvae.

Conversely, the adult germline was found to be sensitive to dietary conditions. Adult males (Fig 5C, ***(3)***), raised from eggs on Standard medium, were collected 1–4 days after eclosion and allowed to mate with *C*(*1*)*DX* virgin females on Standard food for one day; the female progeny (***(4)***) of this cross served as “Control” (i.e., time-zero) datum for subsequent comparisons. The next day, the males were transferred to experimental conditions (Std, SY10, or SY30), with or without rapamycin, and were allowed to feed. Males (***(5)***) were crossed with fresh *C*(*1*)*DX* virgins after 20 days. In this way, we were able to sample the germline of the same group of males before and after treatment (Fig 5C).

**Fig 6:**
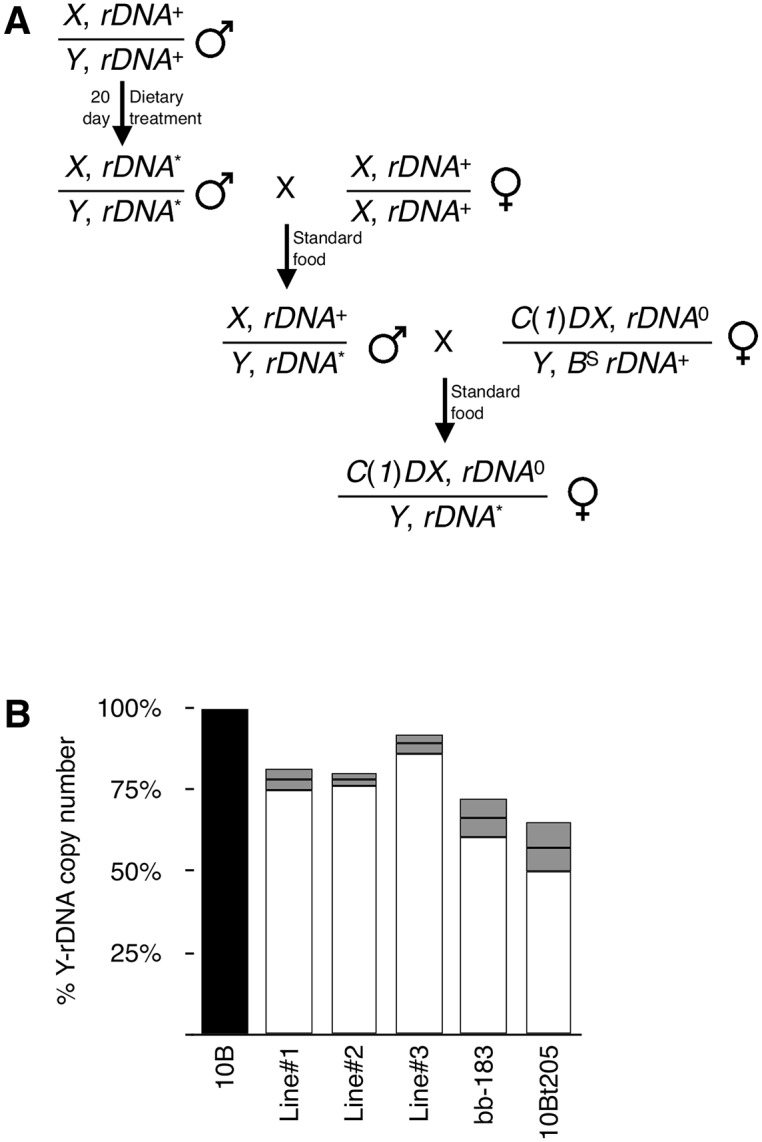
rDNA deletions persist through multiple generations. **(A)** Crossing scheme used to establish long-term rDNA deletion stocks and to isolate *Y*-rDNA arrays for real-time PCR analysis. Nomenclature is as in Fig 5. **(B)** *Y*-rDNA copy number of three independent lines (“Lines” 1–3) established from SY30-fed males. *Y*-rDNA was isolated and quantified two generations after dietary treatment. Percentages calculated relative to *Y*-rDNA isolated from F0 males (top line in (A)) prior to treatment. *Y*-rDNA copy number of an *I*-*Cre*I induced rDNA deletion (“bb-183”) no fewer than sixty generations after it was established, and subsequently maintained on Standard media. *Y*-rDNA copy number of a mutation-induced rDNA deletion (“10Bt205”) approximately 25 generations after it was established, and subsequently maintained on Standard media. Percentage calculated relative to the control line from which the deletion stock was generated. DNA was isolated from pools of ten sibling females and error bars represent standard error of the mean of three technical replicates.

Female progeny (“Treated” in Fig 5C, ***(6)***) had no fewer *Y*-linked rDNA than their half-sisters if their fathers had spent 20 days eating Standard food (Fig 5D, compare “Control” and “Std”). In stark contrast, daughters of fathers who ate SY10 or SY30 for 20 days exhibited a reduced *Y*-rDNA copy number, which was different from daughters both of young fathers and fathers who had fed on Standard food for 20 days. rDNA copy number reduction was greater from SY30-fed fathers than from SY10-fed fathers. Thus the father’s germline underwent loss of rDNA in pre-meiotic mitotic (stem-cell) divisions during the 20 day period that they were exposed to SY10 or SY30, and the amount of loss was proportional to dietary largess.

Loss was mitigated when 10 µM rapamycin was included in the SY10 and SY30 food. It has previously been confirmed that rapamycin concentrations up to 200 μM have no effect on Drosophila feeding rates [72] suggesting that the effects of rapamycin on germline rDNA loss are pharmacological in origin as opposed to behavioral. Spermatogenesis and germline stem cell proliferation in adults are regulated by both diet and nutrient sensing pathways [73, 74]; it is likely that germline rDNA instability in response to increased dietary yeast concentration is a result of the modulation of these pathways, along with any subsequent changes in rDNA regulation.

### Altered rDNA copy number can be stable for many years

In order for a population to maintain a steady-state rDNA size, natural loss must be balanced by expansion. In Drosophila, rDNA magnification may serve this purpose, although magnification is not wide-spread and is only observed on some chromosomes under certain conditions [30–32, 75]; the determinative characteristics of chromosomes that exhibit magnification remain unknown. To test for this sort of reversion of diet-induced rDNA loss, we established independent lines from SY30-fed males and kept them on Standard food as with any Drosophila strain (Fig 6A). This allowed us to monitor transgenerational rDNA copy number for reversion or continued instability. We tested pooled males from three such independent lines that had been kept for two generations on Standard food after being raised for one generation and 20 days as adult on SY10 or SY30 and found that lost rDNA remained lost. Data shown in Fig 6B (Lines 1, 2, and 3) show standard error of the mean (S.E.M.) to report the average rDNA copy number of individuals line founded by single males. This observation is consistent with published findings (as well as our anecdotal observations) that while some engineered rDNA deletions lines exhibited moderate (~5%) expansion shortly after production [55], they have been otherwise stable, without selection, over many subsequent generations. Indeed, we tested one such line and found that in relation to the progenitor stock, changes—magnification or continued loss—had not occurred after six years on Standard food, corresponding to no fewer than sixty generations (Fig 6B, bb-183). Loss of rDNA was also achieved by passaging the *Y*,*10B* in a mutant heterozygous for *Su*(*var*)*205*, which encodes the Heterochromatin Protein 1 gene product. This mutation has been shown to cause rDNA instability [23] and rDNA loss [57], but when the *Y*,*10B* “tempered” by the mutation (*10Bt205*) was returned to a genetic background wild-type for *Su*(*var*)*205*, and passaged for a year (approximately 25 generations) the rDNA loss was not reverted (Fig 6C). These observations suggest that, just like any other polymorphism, rDNA deletions (naturally-occurring or otherwise) persist over multiple generations and that magnification, in contrast, is relatively rare.

## Discussion

In this work we have established that ribosomal DNA (rDNA) copy number polymorphisms can be created by manipulating the diet of wild-type flies. By directly altering insulin-like signaling and phenocopying nucleolar instability in culture using recombinant insulin, we showed that normal IIS signaling can be a significant source of rDNA copy number variation in the soma. Diet-induced rDNA copy number changes occur in both the soma and germline. As a result, they are both permanent within an organism and are capable of being transmitted to subsequent generations, hence may act as a codex for dietary history of an individual or for a population.

Dietary modulation can account for loss of rDNA, but some unknown factor must be responsible for establishing some limit to the loss. We do not know the mechanism for this maintenance, although it could be an as-yet unobserved intentional regulated processes that assures minimal rDNA copy number, or it could be by normal selective pressures exerted by the Minute or bobbed phenotypes that result from very low ribosome number. Alternatively, loss may be balanced by gain of rDNA through unequal sister chromatid exchange, gene conversion, re-replication, or cycles of excision, rolling-circle replication, and re-integration. Meiotic magnification and somatic pseudo-magnification at the rDNA have long been known in Drosophila, although the identification of a mechanism has eluded researchers for over 40 years [32, 76]. Part of the asymptotic limit to loss may be the natural ecology of Drosophila, wherein older males (with greater loss, Fig 5) may be less likely to mate, produce fewer offspring, or produce an altered sex ratio; ecological experiments would be needed to address these possible contributions.

The rDNA is the major site of nuclear energy utilization—transcription, processing, packaging, and export—and was known to be responsive to the energy status of the cell. This response by rDNA to diet, and its fortuitous cleavage by I-CreI, allowed us to identify rDNA copy number as a factor which stabilizes the genome. This observation is now confirmed in Drosophila [26, 43] and similar hypotheses have been proposed for yeast rDNA [40, 77]. However it seems unlikely that the rDNA is alone in this ability. Half of the genome of Drosophila is composed of interspersed or tandem repeats—the transposable elements, highly-repetitive DNAs, expressed repeat gene clusters—and these sequences may account for some of the remaining regulatory variation that as yet has been unmapped [63]. It will require the ability to alter and measure copy numbers of the other repeated DNAs of the genome to ascertain if complex or quantitative traits map to these large blocks of “junk.”

Our observation of diet-induced rDNA loss integrates with previous results which indicate that rDNA copy number polymorphisms account for a large fraction of *Y*-linked gene regulatory variation (termed “YRV”) [43, 56, 63, 78], including the ability of heterochromatin to induce gene silencing (position effect variegation). Ecological phenotypic variation implied by gene expression differences may be quite significant in competitive, food-rich or food-scarce natural environments. Our observations may directly explain why food and culture conditions alter the extent of position effect variegation, and may further explain why chromosomes from different strains—natural isolates or mutant stocks—differ in their ability to suppress position effect variegation [43].

We believe that the diet- and IIS-induced rDNA instability we observe is a general, or at least common, feature of *Y*-linked rDNA because it has been measurable in males of many strains used in our work. For instance, we specifically tested two other *Y* chromosomes: a wild-type male from a laboratory *Canton-S* stock and a freshly wild-caught (“*Texas-B*”) male by backcrossing males from these strains to a *y* ^1^; *bw* ^1^; *e* ^1^; *ey* ^R^ strain to genetically isolate the *Y* chromosome [63]. rDNA copy number of flies raised on SY10 or SY30 was compared and otherwise-isogenic males bearing the *Canton-S* exhibited a 38% decrease in rDNA copy number, while the *Texas-B* chromosome exhibited an 8% decrease. Thus, while diet-induced loss appears to be a common feature of *Y*-linked rDNA genes, there are likely other genetic factors that influence the rate or bounds of loss. Additionally, the two presumably-unrelated transgenic lines (the *Y* from the UAS-InR strain presented in Fig 3 and the *Y* from the Fibrillarin-RFP strain presented in Fig 4) both showed nucleolar instability under conditions with increased IIS signaling. The same phenomenon of rDNA loss was less clear in females, who appeared to exhibit small amounts of loss that was not statistically robust. Because the biology of *X*-linked rDNA arrays differs from that of the *Y*-linked arrays [71, 79, 80], and the consequence of *X*-*X* exchange at the rDNA is very different from that of *X*-*Y* exchange, we had no reason to believe that the phenomenon was related and pursued it no further.

rDNA instability is observed in a number of eukaryotes and is associated with a variety of complex phenotypes including position effect variegation in Drosophila [24, 43], replicative lifespan in yeast [65], plant size in flax [81], cancer progression in humans [82–84], and the aforementioned “hidden variation” of *Y*-linked Regulatory Variation. Our findings provide a mechanism for the influence of diet on all of these processes. Our findings are likely generally relevant to many organisms due to the conserved structure of ribosomal DNA arrays, the common copy number polymorphisms at that locus [34], and the common modes of rDNA regulation [85]. While we focused on diet, other processes that influence rRNA transcription (e.g. cell proliferation, DNA damage, determination and differentiation, stress, aging, temperature, etc. [86]) would presumably also affect rDNA stability via similar mechanisms, and thus, the rDNA may be a common mediator of induced and heritable effects. We do not expect that induced changes to the genome are limited to the rDNA, in fact satellite sequences show copy number polymorphisms that are only now being investigated [57, 87]. In terms of epigenetic inheritance, it is unclear whether diet-induced rDNA copy number polymorphisms may act as an inducible and heritable modifying mutation that subsequently destabilizes epigenetic silencing.

## Materials and Methods

### Fly strains and husbandry

*y* ^*1*^ *w* ^*67c23*^ */Dp(1;Y) y* ^*+*^, *P{w = RS5}10B* was described previously [55, 88], and was used for all feeding experiments. Constitutively active insulin receptor expression was performed using the following stocks: w*; *P{Ubi-GAL4}2/CyO*, *w* ^*1118*^ *; P{Sgs3-GAL4*.*PD}TP1*, *y* ^*1*^ *w* ^*1118*^ *; P{UAS-InR*.*R418P}2*, and *y* ^*1*^ *w* ^*1118*^ *; P{w[+mC] = UAS-InR*.*K1409A}2* [89], all of which were obtained from the Bloomington Drosophila Stock Center. *w* ^*1118*^ *; P{w[+mC] = UAS-mRFP-Fib}* was used for *in vitro* salivary gland culturing experiments to visualize nucleolar fibrillarin [70], and was a gift from Dr. Patrick DiMario. Expression was under control of the core hsp70 promoter on the pUAST backbone, which provided ample expression to visualize without a GAL4 driver. *C(1)DX*, *y* ^*1*^ *f* ^*1*^ *bb* ^*0*^ was used to genetically isolate *Y* chromosomes for real-time PCR analysis. The rDNA deletion strain used in Fig 2 and 6 for comparison was *y* ^*1*^ *w* ^*67c23*^ */Dp(1;Y) y* ^*+*^, *P{w = RS5}10B*, *Df(rDNA)bb-183* [55], and the *10Bt205* strain in Fig 6 is described in Aldrich and Maggert [57]. All crosses were performed at 25°C and 80% humidity. Unless otherwise noted in the experiment all stocks were maintained on standard cornmeal molasses medium.

### Feeding experiments

We used two experimental media for our feeding experiments. The first was based on SYA media used in dietary restriction studies [52] and contained 5% sucrose, 10% hydrolyzed yeast, 5% cornmeal (w/v), and 1% agar (w/v). These ingredients were boiled in deionized water and mixed until fully dissolved. Media was allowed to cool to 55°C before the addition of propionic acid and tegosept to 0.3% each. We refer to this diet as SY10. SY30 was identical except it contained 30% yeast (w/v). One-third of the required yeast was added in increments during heating to allow easy dissolution. Standard (cornmeal) medium is 5% cornmeal (w/v), 3% yeast (w/v), 1% agar (w/v), 7% molasses (v/v), and supplemented with proprionic acid and tegosept as above.

For experiments testing the effects of larval diet, we collected embryos overnight on apple juice agar plates covered with yeast paste. Embryos were washed off plates using 1X PBS and transferred to experimental media at a uniform density [90]. To measure adult germline effects, males raised on Standard medium were collected 1–4 days post eclosion and crossed in groups of five to *C*(*1*)*DX*/*Y* virgins on Standard medium. After 24 hours, the males were removed and placed on either Standard medium, or Experimental media with or without 10 μM Rapamycin (LKT Laboratories), while females were maintained on Standard medium and allowed to lay eggs. Progeny from these females served as the baseline for subsequent comparisons. Males were transferred to fresh experimental media every 3 days for 20 days, after which they were again crossed to virgin *C*(*1*)*DX* females on Standard medium. Additional adult males fed in the above manner were crossed to *y* ^*1*^ *w* ^*67c23*^ females to establish stocks for subsequent analysis.

### RNA analyses

Expression of the rRNA faces three problems. First, it is very stable (by RNA standards), with a half-life of at least two days [91], so steady-state rRNA levels are insufficient to detect changes in rRNA transcription. Second, it is very abundant, accounting for approximately 50% of transcription and 80–90% of steady-state RNA [20], so small differences in loading result in large variance in apparent rRNA concentration. Additionally, selection of any mRNA as a comparison (“denominator” in relative-abundance calculations) introduces even more variance as the quantification of differences in rRNA is more sensitive than differences in mRNAs with lower abundance (i.e., a 10% difference of 1000 is easier to detect than a 10% difference of 10). Third, the *a priori* assumption that any mRNA may not change in conditions in which rRNA expression changes may not be valid. For these reasons, we measured active pre-rRNA by detecting cDNAs derived from the *ETS*-*18S* junction, using reverse transcription primer GGAGGACGAGAAAATTGACAGACGCTTTGAGACAAGCATATAA. This primer was designed to be complementary to the *18S* at the junction of *ETS* and *18S*, and have a novel sequence at the 5’ end for use in subsequent real time PCR.

RNA and DNA were co-purified to satisfy the need for a stable (non-regulable) denominator for rRNA transcription levels. Total nucleic acids were purified from pools of either one hundred second instar larvae (for Fig 1A) or ten dissected and everted third instar larvae (for measuring effects of rapamycin and actinomycinD). Tissue was homogenized in a solution containing 50 mM EDTA, 100 mM Tris pH 7.0, and 1% SDS. Homogenate was extracted twice in equal volumes phenol:chloroform:isoamyl alcohol (25:24:1, buffered with 1 M Tris pH 7.0). Under these conditions, all nucleic acids partition to the aqueous phase [92], which was further extracted with chloroform followed by diethyl ether. Nucleic acids were precipitated with 0.8 volumes propanol, washed with RNAse-free 70% ethanol, and resuspended in DNAse- and RNAse-free water. Reverse transcription was performed in 20 µL reactions with 2 µg nucleic acid, 2 units M-MuLV Reverse Transcriptase (New England Biolabs), 1X Reverse Transcriptase Buffer, 2 µM of each dNTP, and 4 µM primer for 1 hour at 42°C followed by 10 minutes at 90°C. Samples were diluted 1:250 for subsequent real time PCR reactions, as described below and before [55, 57]. Melt-curve analysis was used to assure single melt peaks, and reaction efficiencies were determined using LinRegPCR [93] (average efficiency for the tRNA gene was 92% and for the *ETS*-*18S* was 82%). Efficiency correction and fold changes were calculated as before [94].

For detection of retrotransposon R1 and R2 expression, RNA was extracted as previously described [95] from three pools of 20 male wandering third instar larvae. Reverse-Transcriptase reactions for *R1*, *R2*, and *rho1* cDNAs were individually primed using CCAGCATACGTATGCTCGCTG (for *R1*), GGGAGTGATTGGAGTTGTTTCCG (for *R2*), or CTAGCGAATCGGGTGAATCCACTG (for *rho1*). Real-time PCR reactions were then performed as below using those same primers and additionally GGGACAGCTTAGTGCACTCTAC (for *R1*), CCCCGGAGTTGCTAATCTAACC (for *R2*), and GTGGAGCTGGCCTTGTGGG (for *rho1*). Negative controls for *R1* and *R2* used the *rho1* cDNA template reaction and *R1* or *R2* real time primer pairs.

### Immunofluorescence and microscopy

Whole mount salivary gland immunofluorescence was performed as in (*11*). Glands were dissected from 3^rd^ instar larvae in 1X PBS and were then transferred to PBT (PBS containing 0.1% Triton X-100) and fixed in PBT containing 3.7% formaldehyde and blocked for 1 hour in PBT supplemented with 1% BSA. Glands were washed and incubated overnight at 4°C with a mouse anti-Fibrillarin primary antibody (Abcam) diluted 1:1000 in PNBT (PBT containing 1% BSA and 500 mM NaCl). Goat anti-mouse conjugated to rhodamine was used as a secondary antibody (1:1000). DNA was counterstained with 1 ng/mL DAPI (MP Biomedicals). All images were obtained using a Zeiss Axioskop 2 epifluorescence microscope running AxioVision (v. 4.6.3.0) with a 20X objective (numerical aperature = 0.5). Sequential excitation was performed at 543 nm (for Rhodamine and RFP) and 405 nm (for DAPI).

### In vitro salivary gland culture

Larvae containing the homozygous *P{w[+mC] = UAS-mRFP-Fib}* were reared in bottles and aged to the early wandering stage of the third instar. Males were dissected in 1X PBS and salivary glands were moved to Schneider media supplemented with 50 mg/mL streptomycin, 50 mg/mL penicillin, and 10% heat-inactivated fetal bovine serum (Gibco). Five to ten glands per condition were co-cultured and treated with 5 µM human insulin (Sigma) for 22–24 hours, then stained with 1 ng/mL DAPI for one hour prior to visualization. For “pre-treatment,” salivary glands were treated with 10 µM rapamycin or 0.6 µM actinomycin D (Acros) for two hours prior to insulin addition. For “post-treatment,” glands were treated with rapamycin or actinomycin D two hours prior to DAPI addition. Images were taken of all stained salivary gland lobes near the anterior end at 20X, post-processed for bright/contrast, and scored for nucleolar structure. A nucleus was determined to contain “multiple” nucleoli if more than one separate focus of fibrillarin fluorescence was discernible. Glands that had no or poor DAPI staining were assumed to be damaged by treatment and excluded from analysis. The entire dissection and culture experiment was performed eight times over the course of two weeks and all data pooled to calculate frequencies presented in Fig 4.

### Quantitative polymerase chain reaction (real-time PCR)

Real-time PCR analysis was performed as described in [55]. DNA was extracted from newly eclosed adult male flies in pools of three or more and quantified using a NanoDrop ND-1000. Real-time PCR was performed with a StepOne Real-time PCR system and Power SYBR Green reagents (Applied Biosciences) using 4 ng DNA per reaction. *18S* rDNA was amplified using primers AGCCTGAGAAACGGCTACCA and AGCTGGGAGTGGGTAATTTAC, while the endogenous loading control, tRNA^K-CTT^, was amplified using CTAGCTCAGTCGGTAGAGCATGA and CCAACGTGGGGCTCGAAC. Relative differences were calculated using the “ΔΔC_T_” method. Each data point presented consists of at least three independent biological samples quantified in triplicated or quadruplicated technical replicates. Error bars generally represent the standard deviation of biological replicates, as justified in the text [96]; error bars are generally asymmetric around the mean (“+” values are higher than “-”values”) because we calculate error on C_T_ values prior to exponential transformation (2^CT^) for presentation. P-values were calculated from ΔΔC_T_ values prior to exponential transformation (to get “fold” values) using StatPlus:mac v. 5.8 (AnalystSoft) or Apple Numbers.
